# Mechanisms Underlying the Antiproliferative and Prodifferentiative Effects of Psoralen on Adult Neural Stem Cells via DNA Microarray

**DOI:** 10.1155/2013/452948

**Published:** 2013-07-30

**Authors:** You Ning, Jian-Hua Huang, Shi-Jin Xia, Qin Bian, Yang Chen, Xin-Min Zhang, Jing-Cheng Dong, Zi-Yin Shen

**Affiliations:** ^1^Institute of Integrated Traditional Chinese Medicine and Western Medicine, Huashan Hospital, Fudan University, Shanghai 200031, China; ^2^Shanghai Institute of Geriatrics, Huadong Hospital, Fudan University, Shanghai 200031, China

## Abstract

Adult neural stem cells (NSCs) persist throughout life to replace mature cells that are lost during turnover, disease, or injury. The investigation of NSC creates novel treatments for central nervous system (CNS) injuries and neurodegenerative disorders. The plasticity and reparative potential of NSC are regulated by different factors, which are critical for neurological regenerative medicine research. We investigated the effects of Psoralen, which is the mature fruit of *Psoralea corylifolia L*., on NSC behaviors and the underlying mechanisms. The self-renewal and proliferation of NSC were examined. We detected neuron- and/or astrocyte-specific markers using immunofluorescence and Western blotting, which could evaluate NSC differentiation. Psoralen treatment significantly inhibited neurosphere formation in a dose-dependent manner. Psoralen treatment increased the expression of the astrocyte-specific marker but decreased neuron-specific marker expression. These results suggested that Psoralen was a differentiation inducer in astrocyte. Differential gene expression following Psoralen treatment was screened using DNA microarray and confirmed by quantitative real-time PCR. Our microarray study demonstrated that Psoralen could effectively regulate the specific gene expression profile of NSC. The genes involved in the classification of cellular differentiation, proliferation, and metabolism, the transcription factors belonging to Ets family, and the hedgehog pathway may be closely related to the regulation.

## 1. Introduction

Adult neural stem cells (NSCs) in the adult nervous system serve as a common source of all neural cells, including neurons, astrocytes, and oligodendrocytes [1]. NSCs persist throughout life in the subgranular zone (SGZ) and the subventricular zone (SVZ) [2] to replace mature cells that are lost during turnover, disease, or injury. NSCs remain relatively quiet under normal circumstances [3]. However, various stimuli, such as blood anoxia, trauma, and oxidative stress, initiate the proliferation and differentiation of NSC [4]. NSCs continually self-renew and expand the pool of undifferentiated cells during early stages. These actively proliferating NSC create neurons and subsequently differentiate into astrocytes and oligodendrocytes. Neurons are the functional components of the nervous system, and they are responsible for information processing and transmission. In contrast, astrocytes and oligodendrocytes are collectively known as glia and play supporting roles that are essential for the proper functioning of the nervous system. Astrocytes have become the focus of brain function research in recent years. Mouse brain astrocytes modulate the excitation of inhibitory neurons and inhibit the general activity of surrounding neurons to prevent the overexcitation of neurons in the nerve ring [5]. Neurons cannot produce an enhanced response without the help of astrocytes [6]. This long-term neuronal reaction is the basis of learning and memory. Astrocytes may aid neurons in the production of this enhanced response for a few hours or several days. Astrocytes may play an important role in brain functional activities.

The plasticity and reparative potential of NSC is regulated by various factors, such as neurotransmitters, growth factors, and other extrinsic factors [7]. Therefore, the factors that control the balance between NSC proliferation and differentiation are critical for neurological regenerative medicine research.

Recent evidence suggests that Chinese traditional medicines protect neural cells, improve the ability to resist the damage, and induce NSC proliferation and differentiation [8]. Notably, some herbs may be involved in the maintenance of NSC, but others induce neurogenesis or astrogenesis. The literature [9] suggests that astragaloside as the main active components in astragalus (Huang-Qi in Chinese) significantly promotes the proliferation of NSCs in vitro. Another evidence [10] has established that the combination of BDNF and salidroside can promote the NSCs in the epileptic brain tissues that differentiate into GABA neurons. Therefore, the use of Chinese medicine and its effective ingredients in NSC research generates a novel direction in nerve regeneration research and demonstrates great potential for the curing of diseases of the central nervous system. 

Psoralens are the linear isomers of the furocoumarin family. Psoralen is extracted from the mature fruit of *Psoralea corylifolia L.* (Bu-Gu-Zhi in Chinese) and exhibit photosensitizing effects and various biological activities. Psoralen is commonly used in combination with long wavelength ultraviolet light for the treatment of a variety of skin diseases, such as psoriasis, vitiligo, cutaneous T-cell lymphoma, pemphigus vulgaris, systemic sclerosis, and systemic lupus erythematosus [11–15]. Varying degrees of remission of these diseases occur after Psoralen treatment. The ability of Psoralen to modulate the proliferation and differentiation of cultured cells in vitro, including epidermal cells [16], vascular smooth muscle cells [17], bladder carcinoma cells [18], mucoepidermoid carcinoma cells [19], mammary cancer cells [20], and osteoblasts [21], has been investigated. Psoralen induces transformation from the G1 phase to the S phase or the G2 phase in osteoblasts, which suggests that these compounds effectively regulate progenitor and stem cells.

A series of experiments were performed to examine the role of Psoralen on the proliferation, multidirectional differentiation of NSC. Microarray libraries were generated from the Psoralen-treated NSC to systematically analyze the transcript profile of Psoralen-treated NSC and gain insight into the underlying molecular mechanisms.

## 2. Materials and Methods 

### 2.1. Animals

Pregnant Kunming female mice were maintained in the animal facility of the Public Health Center of Fudan University. All procedures were approved by the Animal Care and Use Committee of Fudan University in accordance with the guidelines for animal use of the National Institutes of Health.

### 2.2. NSC Preparation and Culture

Neurosphere culture was performed as described previously [22] with some modifications. Mouse embryos at embryonic day 14 (E14) were collected from timed-pregnant Kunming mice and placed in D-PBS (Invitrogen, CA, USA). The forebrain neuroepithelium was removed from the embryos under a dissection microscope. The resultant tissue was dissociated by mechanical dissociation into a single-cell suspension using a small-bore, fire-polished Pasteur pipette. The cells were filtered through a sterile nylon mesh and washed twice with a DMEM/F12 medium (Invitrogen, CA, USA) containing 100 units/mL penicillin and 100 *μ*g/mL streptomycin. The number of viable cells was determined by trypan blue staining. Neurosphere culture was initiated by seeding the cells at a density of 1 × 10^5^ to 2 × 10^5^ viable cells/mL in the basal medium supplemented with 20 ng/mL human recombinant fibroblast growth factor-2 (hrFGF2, Invitrogen, CA, USA), 20 ng/mL human recombinant endothelial growth factor (hrEGF, Invitrogen, CA, USA), and Stempro NSC supplement (Invitrogen, CA, USA). The surface of the culture dishes was coated with Poly-D lysine (PDL) (10 mg/mL, Millipore, USA) to prevent cell attachment.

### 2.3. Neurosphere Formation Assay

Cells were plated under clonal conditions at 5 cells/*μ*L in 96-well plate (0.1 mL/well) in serum-free DMEM/F12 medium containing 20 ng/mL hrFGF-2 (Invitrogen, CA, USA), Stempro NSC supplement (Invitrogen, CA, USA), 100 units/mL penicillin and 100 *μ*g/mL streptomycin. The next day, various concentrations (10 nM, 50 nM, and 100 nM) of Psoralen (Yousi Biotechnology, Shanghai, China; purity above 99% HPLC) were added to each well. The total number of spheres that formed in each well was quantified after 8 d. Only colonies >40 *μ*m in diameter were counted as neurospheres. Neurosphere size was determined by measuring the diameters of individual neurospheres under light microscopy, and it is expressed as a volume (assuming a spherical shape). The consecutive second, third, or fourth passages were used to determine neurosphere formation. 

### 2.4. Cell Proliferation Assay

Cell proliferation was based on the incorporation of EdU and its subsequent detection by a fluorescent azide through a Cu(I)-catalyzed [3 + 2] cycloaddition reaction (“click” chemistry) as described previously [23]. In brief, single NSCs were grown in 96-well plates in DMEM/F12 medium containing 20 ng/mL hrFGF-2 and hrEGF (Invitrogen, CA, USA), Stempro NSC supplement (Invitrogen, CA, USA), 100 units/mL penicillin, and 100 *μ*g/mL streptomycin. EdU was added to the culture media in a final concentration of 10 *μ*M for 3 h. Cells were fixed in formaldehyde and penetrated with 0.5% Triton X-100. The cells were stained during a 30 min incubation with 100 mM Tris, 0.5 mM CuSO_4_, 10 *μ*M Alexa 594-azide, and 50 mM ascorbic acid. Cells were counterstained with 4′-6-diamidino-2-phenylindole (DAPI). The cells were washed and imaged using fluorescence microscopy. 

### 2.5. Differentiation Assay

Single NSCs were plated at a density of 5000 cells/well in 10 *μ*g/mL PDL-coated 96-well culture dishes (Corning, NY, USA) and incubated for 3 d in a differentiation medium of DMEM/F12 containing 1% fetal bovine serum (Invitrogen, CA, USA), Stempro NSC supplement (Invitrogen, CA, USA), 100 units/mL penicillin, and 100 *μ*g/mL streptomycin. The cells were harvested 3 d later for Western blot and immunocytochemical analyses. 

### 2.6. Western Blot Analysis

Cells cultured using the differentiation protocol were harvested and lysed in a buffer containing 50 mM HEPES-NaOH (pH 7.5), 100 mM KCl, 1% Triton X-100, 1% sodium deoxycholate, 0.1% sodium dodecyl sulfate, 1 mM EGTA, 1 mM dithiothreitol, 1 mM phenylmethylsulfonyl fluoride, 0.5% protease inhibitor cocktail (Sigma-Aldrich, MO, USA), 1 mM Na_3_VO_4_, 10 mM NaF, and 20 mM *β*-glycerophosphate. The resultant extracts were centrifuged at 14,000 g for 15 min at 4°C to obtain clear cell lysates. Protein concentrations were determined using the Biotime protein assay kit (Beyotime, Shanghai, China) with BSA as a standard. Equal amounts of 35 *μ*g of proteins were loaded on a sodium dodecyl sulfate polyacrylamide gel and separated by electrophoresis. Separated proteins were transferred to nitrocellulose membranes (Amersham Biosciences, USA). The membranes were blocked with 5% (w/v) skim milk in phosphate-buffered saline containing 0.1% Tween 20 and blotted with antibodies for glial fibrillary acidic protein (GFAP) (1 : 500; Chemicon, USA) and *β*-tubulin III (TuJ1) (1 : 200, Chemicon, USA) followed by incubation with appropriate sets of secondary HRP-conjugated goat anti-mouse or rabbit antibodies (1 : 5000; Jackson ImmunoResearch, USA). Immunoreactive bands were visualized with ECL reagents (Biyotime, Shanghai, China). 

### 2.7. Immunocytochemistry

The cells cultured using the differentiation protocol were fixed for 20 min in 4% paraformaldehyde, blocked in 1% BSA and 0.1% Triton X-100, washed with PBS, incubated for 30 min with 0.3% H_2_O_2_ to inhibit endogenous peroxidases, and blocked for 1 h using 3% BSA in PBS/0.1% Triton X-100. The following primary antibodies were incubated for 2 hours at room temperature: monoclonal rabbit anti-GFAP (1 : 500; Chemicon, USA), mouse anti-TuJ1 (diluted 1 : 200; Chemicon, USA), and rabbit antinestin (1 : 1000, Chemicon, USA). Secondary Alexa-conjugated 594 F(ab)′2 goat anti-rabbit antibody and 488 Alexa-conjugated goat anti-mouse IgG (H + L) (1 : 1000; Invitrogen, USA) were added for 1.5 h in PBS in 1% BSA and 0.1% Triton X-100. The cells were counterstained with DAPI. The number of immunoreactive cells in each well was quantified using fluorescent microscopy. 

### 2.8. Microarray and Data Analysis

The cells cultured using the differentiation protocol for 3 days were harvested and lysed in TRIzol Reagent (Invitrogen, USA). Total RNA was isolated using the Qiagen RNeasy kit (Qiagen) in accordance with the manufacturer's protocol. The isolated RNA was subject to a quality control test. RNA from each sample was used for cDNA synthesis followed by the labeling of the cDNA with Cy3. The labeled cDNA samples were submitted to NimbleGen and hybridized to Mouse Gene Expression 12x135K Arrays (Roche NimbleGen, 05543797001) that represent 44,170 mouse genes. The single-color NimbleGen arrays were scanned using a GenePix 4000B microarray scanner. The data were extracted from the scanned images using NimbleScan v2.5 Software. Expression data were normalized through quantile normalization, and the Robust Multichip Average (RMA) algorithm was included in the NimbleScan software. The Probe level (∗_norm_RMA.pair) files and Gene level (∗_RMA.calls) files were generated after normalization. All gene level files were imported into Agilent GeneSpring GX software (version 11.5.1) for further analysis. 

### 2.9. Quantitative Real-Time PCR

Total RNA from cells was extracted using TRIzol reagent (Invitrogen, USA). One microgram of total RNA was reverse transcribed using the Advantage RT-for-PCR kit (Qiagen, Valencia, CA). Freshly transcribed cDNA was used for quantitative real-time PCR using SYBR Green (Bio-Bad, Hercules, CA). The primers for each gene were designed using the online tool, Primer3 (http://frodo.wi.mit.edu/), and the sequences are listed in supplementary Table 1. PCR was performed using a RotorGene real-time DNA amplification system (Corbett Research, Sydney, Australia) as described in our previous study [24].

### 2.10. Bioinformatic Analysis

Differentially expressed genes (*P* < 0.05) between the control and Psoralen-treated groups were functionally annotated via the functional annotation tool, Database for Annotation, Visualization, and Integrated Discovery (DAVID) (http://david.abcc.ncifcrf.gov/), which was developed within the Gene Ontology Consortium [25]. The KEGG pathways of the differentially expressed genes between the control and Psoralen-treated groups were also matched using the DAVID functional annotation tool.

The key regulatory processes of the Psoralen effect were analyzed using the Gene Ontology (GO) Tree Machine (http://bioinfo.vanderbilt.edu/webgestalt/) [26]. The directed acyclic graph (DAG) was generated automatically by the GO Tree Machine for the input gene sets, which was created to identify the most important GO categories and suggest their potential biological importance.

Transcription factors are of great significance for cellular differentiation and proliferation. The online tool, oPOSSUM (http://www.cisreg.ca/oPOSSUM/), was used for the analysis of transcription factor binding sites of differentially expressed genes [27].

### 2.11. Statistical Analysis

All data were expressed as the means ± SD. The statistical significance was calculated using One-way ANOVA (analysis of variance) followed by the least significant difference (LSD) test for post hoc analysis. The significance level was defined as *P* < 0.05. The number of replicated experiments is indicated in the results section or the figure legends.

## 3. Results 

### 3.1. Psoralen Inhibited Neurosphere Formation

**Figure 1:**
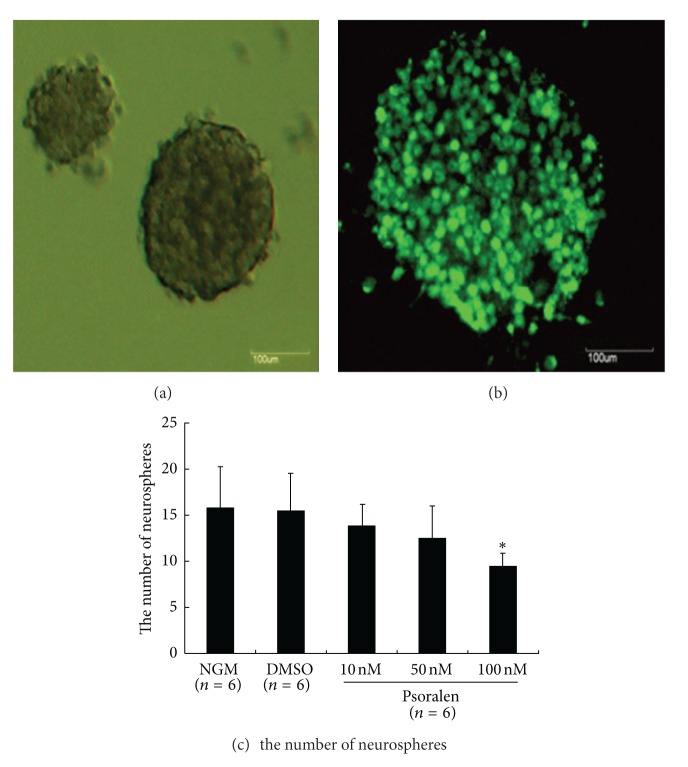
Effects of Psoralen on neurosphere formation of neural stem cells in vitro. Single NSCs at a density of 500 cells/well were cultured in normal growth medium (NGM) containing DMEM/F12 supplemented with hrFGF for 7 days to form various size of neurospheres (a). The neurospheres expressed NSC marker nestin (b). Single NSCs were exposed to NGM, DMSO (0.1%), and 10, 50, 100 nM Psoralen dissolved in DMSO (0.1%), respectively. Psoralen caused a significant decrease in frequency of neurosphere formation (c). Scale bars: 100 *μ*m. Results were expressed as mean ± S.D. of six independent experiments. *P < 0.05 versus NGM.

Neurosphere formation demonstrates the self-renewal ability of NSC when single NSC are plated at a very low cell density. NSC formed neurospheres of various sizes in our growth culturing conditions with diameters ranging from 20 *μ*m to greater than 100 *μ*m (Figure 1(a)). These neurospheres exhibited positive nestin staining (NSC marker), which suggests the presence of NSC and/or neural progenitor cells (Figure 1(b)). We calculated the frequency of neurosphere formation in the presence or absence of Psoralen. The control group formed 15.75 ± 4.43 (*n* = 6) neurospheres from the initially seeded 500 hundred cells (frequency of approximately 3.15%). The solvent (DMSO) did not alter the frequency of neurosphere formation (15.5 ± 3.99, *n* = 6). The neurosphere frequencies for the low (10 nM), middle (50 nM), and high (100 nM) concentrations of Psoralen were 13.91 ± 2.17, 12.56 ± 3.41, 9.43 ± 1.53 (*n* = 6), respectively. The high Psoralen concentration (100 nM) significantly decreased the neurosphere formation of NSC compared to control (*P* < 0.05) (Figure 1(c)). 

### 3.2. Psoralen Inhibited NSC Proliferation

**Figure 2:**
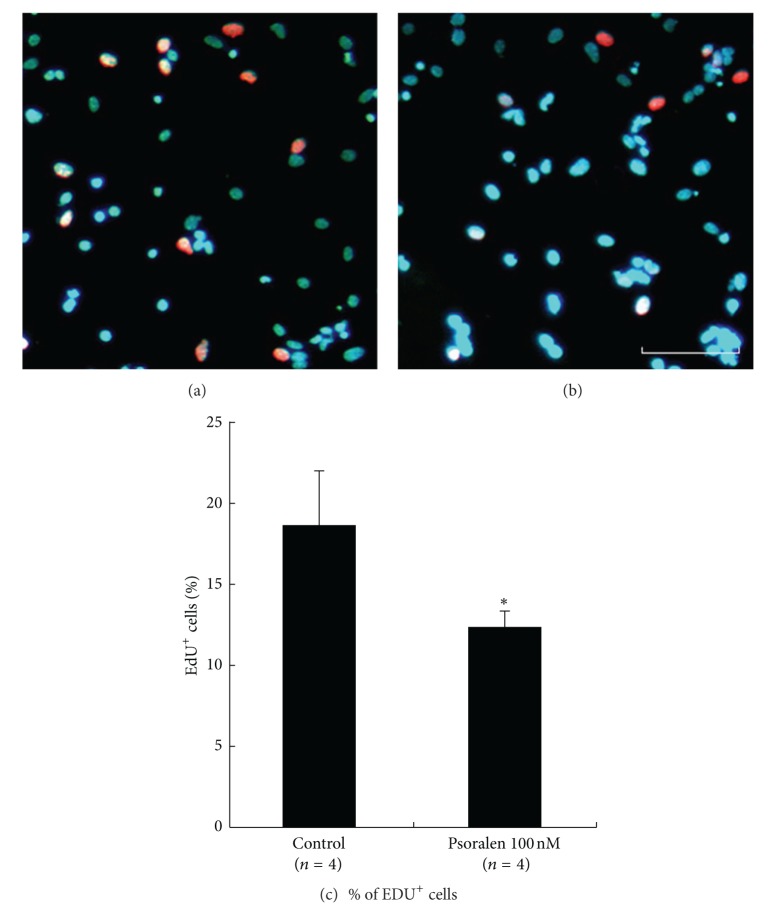
Effects of Psoralen on the proliferation of NSC. Single NSCs were plated at a density of 5000 cells per well in PDL-coated 96-well plate for 12 h. Then, cells were subjected to 10 nM EdU for 2 h, followed by addition of 100 nM OA (b) or not (a). Then Edu immunofluorescence analysis was performed. The cell nuclei were counterstained with DAPI. The percentage of EdU-positive cells in total of 1000 cells was calculated. As a result, Psoralen significantly inhibited the DNA incorporation (c). Scale bars: 100 *μ*m. Results were expressed as mean ± S.D. from four independent experiments. *P < 0.05 versus control.

The decrease in neurosphere formation may have been due to a compromised cell proliferation of NSC. Therefore, we investigated NSC proliferation in the presence of Psoralen using EdU incorporation, which identifies cells in the S phase of the cell cycle. The ratio of EdU-positive cells to total cells was 18.8 ± 3.2% (*n* = 4) in the control group. The incorporation of EdU into NSC decreased significantly in the presence of 100 nM Psoralen (12.6 ± 0.8%, *n* = 4; *P* < 0.05 versus control) (Figures 2(a), 2(b), and 2(c)). 

### 3.3. Psoralen Induced the Differentiation of NSC to Astrocytes

**Figure 3:**
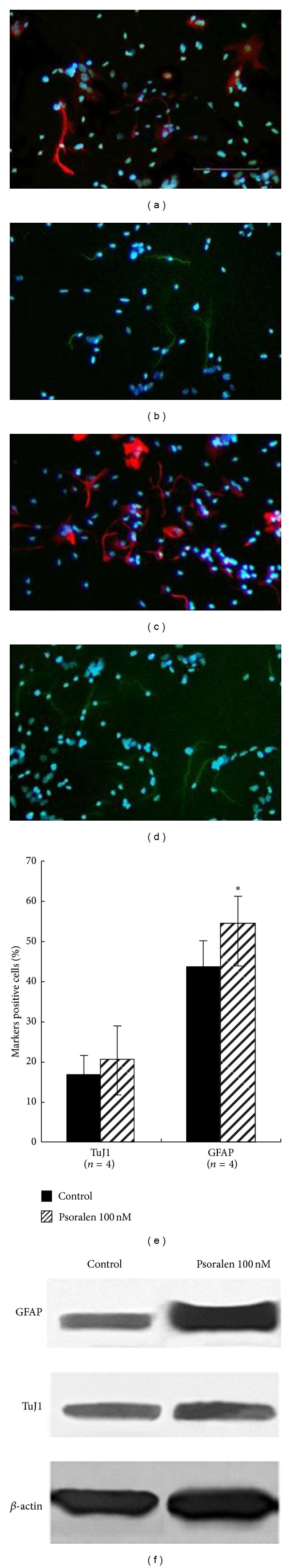
Effects of Psoralen on the differentiation of NSC. Single NSCs were seeded at a density of 50 cells/*μ*L in PDL-coated 96-well plate in differentiation medium for 48 h without Psoralen (a, b) or with 100 nM Psoralen (c, d). Cells were subjected to primary antibodies of GFAP, TuJ1 and corresponding secondary antibodies and visualized with Alexa-conjugated 594 F(ab)′2 goat anti-rabit antibody and 488 Alexa-conjugated goat anti mouse IgG (H + L). The ratio of GFAP, TuJ1-positive cells against DAPI-stained cells was calculated. Psoralen significantly increased the GFAP-positive cells and decreased the TuJ1-positive cells (e). The same cells were performed using Western blotting. Psoralen significantly increased the GFAP, decreased the TuJ1 protein expression (f). Scale bars: 100 *μ*m. Results were expressed as mean ± S.D. from three independent experiments. *P < 0.05 versus control.

Single cells were cultured in a monolayer on PDL-coated dishes in serum without growth factors for 48 h. Immunochemistry revealed that 43.93 ± 6.21% (*n* = 4) of cells in the control group were GFAP-positive (Figure 3(a)) and 18.45 ± 4.6% (*n* = 4) of cells were TuJ1-positive (Figure 3(b)), which suggests multi-potential NSC. The percentage of TuJ1-positive cells did not change after Psoralen treatment (*P* > 0.05 versus control) (Figure 3(c)), but the percentage of GFAP-positive cells significantly increased to 54.32 ± 6.33% (*n* = 4; *P* < 0.05 versus control) (Figure 3(d)). Western blotting analyses of total cell lysates in the Psoralen-treated or untreated cells revealed an increase in GFAP expression. The expression of TuJ1 protein, a neuron-specific marker, was slightly decreased (Figure 3(f)). These results suggested that Psoralen induces the differentiation of NSC to astrocytes. 

### 3.4. Microarray and Data Analysis

We screened the differential expression of genes that were induced by Psoralen using the microarray technique. A total of 129 genes were up-regulated by Psoralen by more than 1.5-fold compared to the control group (*P* < 0.05), and 146 genes were downregulated by more than 1.5-fold (*P* < 0.05).

### 3.5. Confirmation of Differentially Expressed Genes Using Quantitative Real-Time PCR

**Figure 4:**
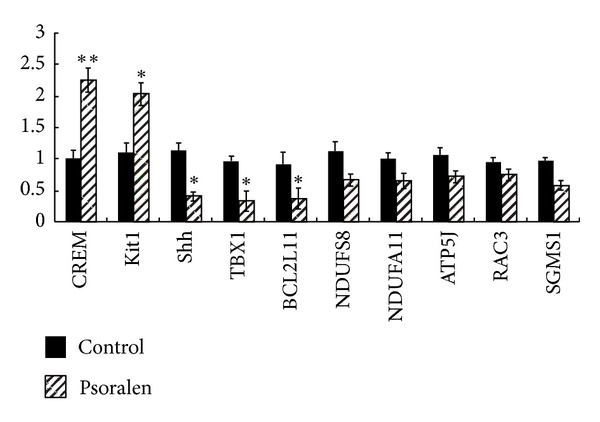
The expression profiles of these representative genes including CREM, Kit1, Shh, TBX1, and Bcl2l11 were confirmed by quantitative real-time PCR, which validates our data. Our data sets could be used for further bioinformatic analyses.

We confirmed the mRNA expression of CREM, Kit1, Shh, TBX1, Bcl2l11 using quantitative real-time PCR, and these genes were chosen based on the gene function after bioinformatic analyses. The results were a highly consistent with the DNA microarray measurements (Figure 4). CREM (cAMP responsive element modulator) encodes a ZIP transcription factor that binds to the cAMP-responsive element in many viral and cellular promoters. Kit1 (Kit ligand), otherwise known as Stem cell factor or Steel factor, is a growth factor important for the survival, proliferation, and differentiation of hematopoietic stem cells and other progenitor cells. Shh (Sonic hedgehog) is a secreted protein that is required to establish patterns of cellular growth and differentiation within ventral regions of the developing CNS. TBX1 (T-box-1) plays an important role in developmental processes. The fourth gene encodes Bcl2l11 (BCL2-like 11, apoptosis facilitator), regulates, and contributes to programmed cell death and apoptosis. The expression profiles of these representative genes using quantitative real-time PCR analysis corresponded to the microarray profiles, which validates our data. 

### 3.6. Comparisons of the DAVID Gene Functional Classification

A total of 276 known genes with different genebank accession numbers were assigned to the data sets using the DAVID Functional Annotation Tool for the functional annotation of the differentially expressed genes. The 261 system-recognized genes were functionally classified into 7 groups using the default settings (medium classification stringency), and the corresponding enrichment scores were greater than 1. These groups included “apoptosis” (12 genes), “regulation of apoptosis” (11 genes), “multicellular organism reproduction” (10 genes), “cellular protein catabolic process” (9 genes), “epidermis development” (4 genes), “cell differentiation” (3 genes), and “regulation of cell cycle process” (3 genes). The details of the differentially expressed genes are listed in Table 1.

### 3.7. DAG Analysis of Gene Categories

DAG visualized enriched gene categories. Ten GO categories were enriched after Psoralen treatment, including “establishment of cell polarity” (2 genes), “myotube differentiation” (2 genes), “positive regulation of angiogenesis” (2 genes), “glycolipid metabolic process” (2 genes), “regulation of pigmentation during development” (2 genes), “negative regulation of DNA metabolic process” (2 genes), “microtubule polymerization or depolymerization” (2 genes), “telomere organization” (2 genes), “mesenchymal cell development” (3 genes), and “neural crest cell differentiation” (3 genes). The details of the differentially expressed genes are listed in Table 2.

### 3.8. Identification of KEGG Pathways

KEGG pathway analysis was assigned using the DAVID annotation tool to reveal the functional roles of the differentially expressed genes. Twenty-nine KEGG pathways were matched to the differentially expressed tags of the two groups, such as “Hedgehog signaling pathway”, “Oxidative phosphorylation”, and “Wnt signaling pathway”. The details of the differentially expressed genes are listed in Table 3. The Hedgehog signaling pathway is a key regulator of animal development, and it also plays an important role in the development of adult NSC. Oxidative phosphorylation is an important metabolic pathway for cellular respiration, glycolysis, and the Krebs cycle. The Wnt signaling pathway plays a key role in cellular differentiation, apoptosis, and development.

### 3.9. Identification of Transcription Factor Binding Sites

oPOSSUM is a web-based system for the detection of overrepresented transcription factor binding sites in the promoters of gene sets [27]. A total of 171 transcription factors were identified in this study, including the gene families of “Ets” “BetaBetaAlpha-zinc finger,” “TATA-binding,” “Helix-Loop-Helix,” and “Hormone-nuclear Receptor”. The details of the differentially expressed genes are listed in Table 4. The transcription factor, Sfpi1_1(PU.1), targeted 139 genes, which may encode an ETS-domain transcription factor that is critical for the determination of cell lineage and the regulation of differentiation versus stem cell proliferation [28]. Recent research indicates that Sfpi1_1 controls dendritic cell development in a dose-dependent manner [29].

## 4. Discussion

NSCs open a new way of treatment of the injured central nervous system and neurodegenerative disorders. Potential uses of NSC in repair include transplantation to repair missing cells and the activation of endogenous cells to provide “self-repair.” Before the full potential of NSC can be realized, we need to know what controls their proliferation and differentiation. Precise control of proliferation and differentiation of multipotent NSC is crucial for proper development of the nervous system. Chinese herb extracts as effective factors regulating the self-renewal and differentiation capacity of NSC have been widely studied [8]. Psoralen can modulate the proliferation and differentiation of a variety of stem cells through different mechanisms [19–21]. 

In the present study, single cells derived from dissected neural tissue form floating balls of cells, termed neurospheres, when plated under nonadherent, permissive conditions [30]. These highly heterogeneous structures contain neural stem cells, more restricted progenitors, and differentiated progeny. The more restricted progenitors and differentiated progeny exhibit limited proliferation capacity, and these cells only form very small neurospheres. However, the neural stem cells demonstrate strong self-renewal and generate neurospheres in continuous passages. The self-renewal potential of cells within neurosphere cultures may be investigated by the dissociation of single cells that must be plated at a density at which a single cell gives rise to a single neurosphere. This process is termed clonal analysis. Single cells that are plated under medium to high cell densities permit the adherence of cells or small neurospheres to each other, and these structures combine to form larger neurospheres [31, 32]. Clonal analysis is impossible in this situation, and the true proliferative capacity of stem cells cannot be properly determined. However, drug-screening assays do not require that each neurosphere derived from a single stem or progenitor cell. A low plating cell density of 5000 cells/mL or 1000 cells/mL is sufficient to measure the self-renewal of neural stem cells [33, 34].

We plated single cells at a density of 5000 cells/mL in 100 *μ*L/well in a 96-well plate. Approximately 5% of the plated single cells reformed neurospheres, which is consistent with previous studies. Psoralen significantly inhibited neurosphere formation with maximum effects at 100 nM. This Psoralen-induced decrease in neurosphere formation may be derived from the compromised proliferation of NSC. Therefore, we investigated NSC proliferation in the presence of Psoralen using an EdU incorporation assay. Psoralen significantly reduced EdU incorporation. These results suggested that Psoralen inhibits the self-renewal of NSC through the inhibition of proliferation. 

NSCs generally and gradually exit the cell cycle when they enter the process of differentiation. The length of the neural progenitor cell cycle is directly coupled to cell fate choices because factors that shorten the cell cycle inhibit differentiation divisions, but factors that lengthen the cell cycle promote differentiation divisions. We also investigated the effect of Psoralen on NSC differentiation. Psoralen increased the percentage of GFAP-positive cells, but the percentage of TuJ1-positive cells was not altered. Psoralen increased GFAP protein expression on Western blots, but it did not influence TuJ1 protein expression. Our experiment demonstrated that Psoralen strongly induced the differentiation of NSC into astrocytes.

A cluster analysis was performed on these differentially expressed genes using DAVID bioinformatics resources. DAVID functional classification suggested that Psoralen primarily altered the expression of genes involved in differentiation, proliferation, apoptosis, and catabolism. The DAG analysis suggested that the effect of Psoralen on neural stem cells is a biological process that impacts the metabolism of DNA, sugar, and lipids and regulates cellular proliferation, split polarization, and differentiation.

There are several genes of interest in the DAVID and DAG classification, including BCL2-like 11, Kit ligand, and Sonic hedgehog, which were confirmed by our previous quantitative real-time PCR test. BCL2-like 11 protein is encoded by a gene in the BCL-2 protein family, which play important roles in the regulation of neuronal and lymphocytic apoptosis. BCL-2 deficiency does not affect neuronal numbers in the cerebellum [35], but this protein has been implicated in hippocampal neuronal protection in a seizure model [36]. BCL-2-deficient mice exhibit relatively normal development of the nervous system, but BCL-2 is important for the postnatal maintenance of specific neuronal subsets, including facial motor neurons and dorsal root ganglia [37]. Kit ligand, also known as stem cell factor (SCF), steel factor (SLF) and mast cell growth factor (MGF), is as 30-kDa glycoprotein with broad activity in various tissues [38]. Kit ligand regulates the development of cellular lineage by expressing c-kit, which affects proliferation and maturation [38, 39]. Kit signaling in the CNS influences oligodendrocyte precursors prior to differentiation towards a myelinated phenotype [39]. Greater than 93% of nestin-positive NSC from the embryonic rat cortex express Kit ligand [40]. Kit ligand is a survival factor for NSPCs during the early stages of differentiation [41]. The Sonic hedgehog (Shh) gene is one of a homolog gene of Hedgehog (hh) [42]. Shh gene plays an important role in the development of the mammary glands, prostate, lung, hair, nervous system, and other organs. Shh regulates the development of the neural system, especially the proliferation and differentiation of neural stem cells [43, 44]. Shh is a positive regulator of adult hippocampal neural stem cell proliferation, and it may participate in injury remodeling.

We further searched the possible transcription factor binding sites existing in the upstream sequences of differentially expressed genes. Consequently, 139 genes differentially expressed were predicted to have transcription factor binding sites for Sfpi1, a member of the Ets family. The common character of Ets factors was highly conserved and the DNA binding domain can combine with specific gene sequence to regulate the expression and function of target gene by being involved in cell proliferation, differentiation, apoptosis, and mesenchymal-epithelial interactions [45, 46]. ETs could be involved in many physiological and pathological processes. A large number of researches showed that Ets transcription factors play an important regulation role in the development, tumor invasion, and metastasis of amphibians, birds and mammals [47].

Sfpi1, also called PU.1, belongs to the SpI subfamily of the Ets family. The transcription factors Sfpi2, Sfpi3 also belong to the same family. These members all showed the existence of the Ets structure domain in the C-terminal of the protein and an acid transcription activation structure domain in the N-terminal. Literature study reported that this kind of structure played an important role in the differentiation process of a variety of cells. The changes of the expression quantity of the transcription factors played a decisive role in adjusting the differentiation direction of stem cells [48]. Sfpi1 was highly expressed in myeloid cells but not in lymphocyte. It functioned in the early state of differentiation in granulocyte, red blood cells, and megakaryocyte, and it suppressed red blood cell differentiating to red blood cells. Studies [28] have shown that a high level of Sfpi1 could cause differentiation of macrophage but not proliferation of progenitor cells. In embryogenesis process, lack of Sfpi1 could lead to development delay [49, 50]. In recent years, study [29] showed that Sfpi1 also played an essential role in differentiation of dendritic cells. Based on bioinformatical analysis and the literature review, we thought that Sfpi1 may mediate the effects of Psoralen. 

The KEGG pathway analysis demonstrated that the effects of Psoralen on neural stem cells were closely related to the Hedgehog signaling pathway, Wnt signaling pathways, MAPK, spliceosomes, axon guidance, oxidative phosphorylation, adherens junction, gap junction, and tight junction. The matched Hedgehog signaling pathway is of interest, which is crucial for the generation and maintenance of both embryonic and adult stem cells. This pathway regulates spinal dorsal-ventral plasticity and the multizone of neural precursor cell proliferation and differentiation in the developing nervous system. Hedgehog signaling pathway also plays a key role in the regulation of neural progenitor cell growth [51]. Granule cell precursor proliferation in the cerebellum requires Hedgehog signaling [52]. Hedgehog signaling may regulate the self-renewal of progenitor cells in the SVZ, hippocampus, olfactory bulb, and other brain areas [51, 53]. The downstream target gene of the Hedgehog pathway, BMI1, regulates central and peripheral nervous system progenitor cell self-renewal [54]. The oligodendrocyte generation of glial precursor cells and the subsequent differentiation into mature oligodendrocytes require Hedgehog signaling in SHH^(−/−)^ mutants [55]. Cell proliferation and differentiation culture conditions in the NSC clones express mRNA of the functional Shh receptor, PTCH, and Shh (5~50 nmol/L) in vitro promotes the proliferation of fetal rat spinal cord in primary and passaged neural stem cells in a dose-dependent manner. This activation is also observed in quiescent adult neural stem cells in which the Hedgehog signal pathway stimulates continuous proliferation and differentiation [56].

Recently, Crosstalk of Hedgehog with other critical signaling pathways has been mentioned. Hedgehog and Wnt pathways resulted in opposite proliferative outcomes of neural stem/progenitor cells, and Sfrp-1 and Gli3 contributed to this negative cross-regulation [57]. But Hedgehog and Notch signaling could cooperate to regulate neurogenic divisions of neocortical progenitors. The transcription factors Hes1 and Blbp were possibly the key molecules in the Hh/Notch co-regulation of corticogenesis [58].

In conclusion, our study demonstrated the antiproliferative and prodifferentiative effects of Psoralen on NSC. The microarray study demonstrated that the gene expression profile of NSC could be specifically regulated by Psoralen. The genes involved in the classification of cellular differentiation, proliferation, and metabolism, the transcription factors belonging to Ets family, and the hedgehog pathway may play important roles in the regulation process, which need further studies.

## Figures and Tables

**Table 1:** The list of the differentially expressed tags of DAVID gene functional classification.

Functional annotation clustering	Gene number	Gene bank accession	Description
Apoptosis	12	BC058175	BCL2-like 11 (apoptosis facilitator)
		AK144648	CASP2 and RIPK1 domain containing adaptor with death domain
		AK186862	Cytokine-induced apoptosis inhibitor 1
		AK049134	Ectodysplasin A2 isoform receptor
		AK010878	Hypothetical protein LOC100233175
		AK041961	Kit ligand
		XM_001476612	Predicted gene 15753, Sp110 nuclear body protein
		BC016606	Proteasome (prosome, macropain) assembly chaperone 2, similar to Clast3 protein
		BC063087	Sonic hedgehog
		AK082974	Sphingomyelin synthase 1
		BC027335	Tectonic family member 3
		AK183781	Zinc finger, DHHC domain containing 16

Regulation of apoptosis	11	BC058175	BCL2-like 11 (apoptosis facilitator)
		AK144648	CASP2 and RIPK1 domain containing adaptor with death domain
		AK186862	Cytokine-induced apoptosis inhibitor 1
		BC080315	Glutamate receptor, metabotropic 7
		BC152841	Heat shock protein 1B, heat shock protein 1A, heat shock protein 1-like
		AK010878	Hypothetical protein LOC100233175
		AK041961	Kit ligand
		AK160757	Microphthalmia-associated transcription factor
		XM_001476612	Sp110 nuclear body protein
		BC016606	Proteasome (prosome, macropain) assembly chaperone 2; similar to Clast3 protein
		AK082974	Sphingomyelin synthase 1

Multicellular organism reproduction	10	BC058175	BCL2-like 11 (apoptosis facilitator)
		AK086849	*beta*-1,4-N-Acetyl-galactosaminyl transferase 1
		AY738720	cAMP responsive element modulator
		BC152841	Heat shock protein 1B, heat shock protein 1A, heat shock protein 1-like
		AK160943	Heterogeneous nuclear ribonucleoprotein L-like, glutathione peroxidase 4
		AK041961	Kit ligand
		AK078529	Neuralized homolog 1A (Drosophila); similar to neuralized 1
		XM_001474694	Rik protein
		BC115962	Germ cell-less homolog 1
		BC061175	Testis-specific serine kinase 2

Cellular protein catabolic process	9	BC058593	SUMO1/sentrin specific peptidase 7
		AK053749	WW domain containing E3 ubiquitin protein ligase 2
		BC040367	Ligand of numb-protein X 1
		BC128498	Myosin, heavy polypeptide 9, nonmuscle
		BC115962	Germ cell-less homolog 1
		XM_001474694	Rik protein
		XM_354829	Seven in absentia homolog 3 (Drosophila)
		BC059027	Similar to midline 1, midline 1
		BC026983	Ubiquitin specific peptidase 39

Epidermis development	4	BC152326	Involucrin, RIKEN cDNA 1110019C06 gene
		BC148438	Small proline-rich protein 2E, small proline-rich protein 2F, Small proline-rich protein 2I
		BC156163	Small proline-rich protein 2H
		BC063087	Sonic hedgehog

Cell differentiation	3	AK041961	Kit ligand
		AF349658	Similar to T-box 1; T-box 1
		BC063087	Sonic hedgehog

Regulation of cell cycle process	3	AK018243	ZW10 homolog (Drosophila), centromere/kinetochore protein
		BC016606	Proteasome (prosome, macropain) assembly chaperone 2, similar to Clast3 protein
		AK003451	Timeless interacting protein

**Table 2:** The list of the differentially expressed tags of DAG analysis of gene categories.

Categories	Gene bank accession	Description
Establishment of cell polarity	17886	Myosin, heavy polypeptide 9, nonmuscle
	20423	Sonic hedgehog
Myotube differentiation	17886	Myosin, heavy polypeptide 9, nonmuscle
	20423	Sonic hedgehog
Positive regulation of angiogenesis	14461	GATA binding protein 2
	12394	Runt-related transcription factor 1
Glycolipid metabolic process	14421	*beta*-1,4-N-Acetyl-galactosaminyl transferase 1
	12916	cAMP responsive element modulator
Regulation of pigmentation during development	12125	BCL2-like 11 (apoptosis facilitator)
	17311	Kit ligand
Negative regulation of DNA metabolic process	66131	Timeless interacting protein
	72836	Protection of telomeres 1B
Microtubule polymerization or depolymerization	17318	Midline 1
	17997	Neural precursor cell expressed, developmentally downregulated gene 1
Telomere organization	15511	Heat shock protein 1B
	72836	Protection of telomeres 1B
Mesenchymal cell development	21380	T-box 1
	20423	Sonic hedgehog
	17311	Kit ligand
Neural crest cell differentiation	21380	T-box 1
	20423	Sonic hedgehog
	17311	Kit ligand

**Table 3:** The list of the differentially expressed tags of matched KEGG pathway analysis.

KEGG pathways	Gene number matched	Gene bank accession	Description
MAPK signaling pathway	5	BC119041	RAS-related C3 botulinum substrate 3
		BC052705	Dual specificity phosphatase 8
		BC152841	Heat shock protein 1B
		BC006037	Mitogen-activated protein kinase kinase kinase kinase 3
		BC054533	Protein kinase, cAMP dependent, catalytic, beta
Pathways in cancer	5	BC119041	RAS-related C3 botulinum substrate 3
		AK041961	Kit ligand
		AK160757	Microphthalmia-associated transcription factor
		AK155262	Runt-related transcription factor 1
		BC063087	Sonic hedgehog
Spliceosome	4	AK040680	Cell division cycle 40 homolog (yeast)
		BC152841	Heat shock protein 1B
		BC004793	Poly(rC) binding protein 1
		BC026983	Ubiquitin specific peptidase 39
Axon guidance	4	AK089130	Eph receptor A5
		BC119041	RAS-related C3 botulinum substrate 3
		AK171860	Actin-binding LIM protein 1
		AK053689	Netrin G1
Hedgehog signaling pathway	3	AK149310	Casein kinase 1, gamma 1
		BC054533	Protein kinase, cAMP dependent, catalytic, beta
		BC063087	Sonic hedgehog
Viral myocarditis	3	BC119041	RAS-related C3 botulinum substrate 3
		BC128498	Myosin, heavy polypeptide 9, nonmuscle
		BC156199	v-abl Abelson murine leukemia viral oncogene homolog 2
Melanogenesis	3	AK041961	Kit ligand
		AK160757	Microphthalmia-associated transcription factor
		BC054533	Protein kinase, cAMP dependent, catalytic, beta
Oxidative phosphorylation	3	AK078484	ATP synthase
		XM_895878	NADH dehydrogenase (ubiquinone) 1 alpha subcomplex 11
		AK149674	NADH dehydrogenase (ubiquinone) Fe-S protein 8
Cytokine-cytokine receptor interaction	3	AK049134	Ectodysplasin A2 isoform receptor
		AY221616	Interleukin 15 receptor, alpha chain
		AK041961	Kit ligand
Cysteine and methionine metabolism	2	AK085987	5-Methyltetrahydrofolate-homocysteine methyltransferase
		BC094469	Mercaptopyruvate sulfurtransferase
Prion diseases	2	BC152841	Heat shock protein 1B
		BC054533	Protein kinase, cAMP dependent, catalytic, beta
SNARE interactions in vesicular transport	2	AK030538	Vesicle transport through interaction with t-SNAREs homolog 1A
		BC086925	Vesicle-associated membrane protein 5
Adherens junction	2	AK137694	LIM domain only 7
		BC119041	RAS-related C3 botulinum substrate 3
Gap junction	2	AK031305	Guanylate cyclase 1, soluble, alpha 3
		BC054533	Protein kinase, cAMP dependent, catalytic, beta
Systemic lupus erythematosus	2	BC152885	Histone H4
		K02799	Complement C4 precursor
Vascular smooth muscle contraction	2	AK031305	Guanylate cyclase 1, soluble, alpha 3
		BC054533	Protein kinase, cAMP dependent, catalytic, beta
Natural killer cell mediated cytotoxicity	2	BC119041	RAS-related C3 botulinum substrate 3
		AY860969	Natural killer cell receptor Ly49C
Parkinson's disease	2	AK078484	ATP synthase
		AK149674	NADH dehydrogenase (ubiquinone) Fe-S protein 8
Tight junction	2	BC002003	Claudin 1
		BC128498	Myosin, heavy polypeptide 9, non-muscle
Ubiquitin mediated proteolysis	2	AK053749	WW domain containing E3 ubiquitin protein ligase 2
		BC059027	Midline 1
Insulin signaling pathway	2	BC054533	Protein kinase, cAMP dependent, catalytic, beta
		AK172276	Regulatory associated protein of MTOR, complex 1
Wnt signaling pathway	2	BC119041	RAS-related C3 botulinum substrate 3
		BC054533	Protein kinase, cAMP dependent, catalytic, beta
Purine metabolism	2	AK031305	Guanylate cyclase 1, soluble, alpha 3
		AK036090	Phosphodiesterase 8B
Alzheimer's disease	2	AK078484	ATP synthase
		AK149674	NADH dehydrogenase (ubiquinone) Fe-S protein 8
Chemokine signaling pathway	2	BC010478	Hemopoietic cell kinase
		BC054533	Protein kinase, cAMP dependent, catalytic, beta
Huntington's disease	2	AK078484	ATP synthase
		AK149674	NADH dehydrogenase (ubiquinone) Fe-S protein 8
Regulation of actin cytoskeleton	2	BC119041	RAS-related C3 botulinum substrate 3
		BC128498	Myosin, heavy polypeptide 9, non-muscle
Neuroactive ligand-receptor interaction	2	AK050472	Gastric inhibitory polypeptide receptor
		BC080315	Glutamate receptor, metabotropic 7
Olfactory transduction	2	BC104278	Olfactory receptor 1173
		BC054533	Protein kinase, cAMP dependent, catalytic, beta

**Table 4:** The list of the differentially expressed tags of matched transcription factor binding sites.

Transcription factor	Gene family	Target gene (>70 hits)
Sfpi1_1	Ets	139
Elf3_1	Ets	131
Zfp105_1	Beta-Beta-Alpha-zinc finger	114
Tbp_1	TATA binding	110
Foxj1_1	Forkhead	100
Sox12_1	High-mobility group	95
Tcfap2a_1	Helix-Loop-Helix	94
Zic2_2	Beta-Beta-Alpha-zinc finger	93
Irf3_1	IRF	92
Foxj3_2	Forkhead	91
Zic3_2	Beta-Beta-Alpha-zinc finger	89
Sox11_1	High-mobility group	89
Sox4_1	High-mobility group	86
Klf7_1	Beta-Beta-Alpha-zinc finger	85
Arid5a_1	Arid	83
Ehf_1	Ets	83
Sox11_2	High-mobility group	80
Sox18_1	High-mobility group	80
Osr1_2	Beta-Beta-Alpha-zinc finger	79
Sox5_1	High-mobility group	79
Sp4_1	Beta-Beta-Alpha-zinc finger	77
Zscan4_2	Beta-Beta-Alpha-zinc finger	76
Tcfap2e_1	Helix-Loop-Helix	76
Egr1_2	Beta-Beta-Alpha-zinc finger	75
Foxl1_2	Forkhead	75
Foxa2_1	Forkhead	75
Tcfap2c_1	Helix-Loop-Helix	75
Sox14_2	High-mobility group	75
Smad3_1	MH1	75
Sox17_1	High-mobility group	74
Hic1_2	Beta-Beta-Alpha-zinc finger	73
Sox21_2	High-mobility group	72
Zic1_2	Beta-Beta-Alpha-zinc finger	71
Tcf1_2	Homeo	71
Zfp740_1	Beta-Beta-Alpha-zinc finger	70
